# In vitro formation kinetics of two 2,5-dimethylfuran phase-I metabolites: *cis*-3-hexene-2,5-dione and 5-methylfurfuryl alcohol

**DOI:** 10.1007/s00204-026-04442-8

**Published:** 2026-05-20

**Authors:** Jonas Appel, Carolin Kulosa, Nico Becker, Simone Stegmüller, Sullivan Sadzik, Elke Richling

**Affiliations:** Department of Chemistry, Division of Food Chemistry and Toxicology, RPTU University Kaiserslautern-Landau (RPTU), Kaiserslautern, Germany

**Keywords:** 2,5-Dimethylfuran, Phase-I metabolism, Human liver microsomes, Cytochrome P450, HPLC–ESI–MS/MS, GC–MS

## Abstract

2,5-Dimethylfuran (DMF) is a heat-induced contaminant found in various thermally processed foods. Due to its structural similarity to furan, a well-known hepatotoxin and potential human carcinogen, the presence of DMF in food poses a potential risk to consumers. DMF undergoes cytochrome P450 (CYP)-mediated biotransformation leading to the formation of two primary phase-I metabolites: the reactive *cis*-3-hexene-2,5-dione (HDO), which can react with cellular nucleophiles, and the primary alcohol 5-methylfurfuryl alcohol (MFA), formed after hydroxylation of the alkyl moiety. To deduce the in vitro formation kinetics of these two phase-I metabolites, we utilised a newly developed HPLC–ESI–MS/MS method to quantify HDO after scavenging with glutathione and monitored the formation of MFA by GC–MS in parallel. Metabolic activation and the formation of HDO was the predominant biotransformation pathway in human liver microsomes, whereas the parallel formation of MFA was significantly less efficient. In line with data on the metabolic activation of furan, CYP2E1 was the most active human CYP-isoform in the potential metabolic activation of DMF. However, CYP3A4 and CYP2D6 also contributed to the HDO formation in vitro. Hydroxylation of DMF and formation of MFA were exclusively catalysed by CYP2E1. Despite the formation of MFA as an alternative metabolic pathway, our kinetic data indicate that DMF is primarily metabolised by CYP2E1 to the reactive *cis*-enedial intermediate HDO, particularly at physiologically relevant concentrations. Therefore, exposure to DMF may contribute to the overall risk associated with dietary exposure to furans.

**Supplementary Information:**

The online version contains supplementary material available at 10.1007/s00204-026-04442-8.

## Introduction

The heat-induced food contaminant furan is categorised as hepatotoxic and possibly carcinogenic to humans (IARC 2B) and has been the subject of intensive research in recent years, focusing on its occurrence in food, human metabolism, and toxicity (NTP 1993; International Agency for Research on Cancer, 1995). Furan formation during thermal treatment of foods is frequently accompanied by the formation of different methylfurans, such as 2,5-dimethylfuran (DMF), which have received less attention in toxicological research to date (Fromberg et al. 2014; Becalski et al. 2016; Condurso et al. 2018; Lipinski et al. 2024). Due to their structural similarities and reported co-occurrence in various food products the European Food Safety Authority (EFSA) concluded that methylfurans, especially 2-methylfuran and 3-methylfuran, might contribute significantly to overall furan exposure and an increased concern for furan-mediated hepatoxicity (Knutsen et al. 2017). The assumed dose additivity is particularly relevant for older population groups whose dietary exposure is mainly attributed to relatively high levels of furan and methylfurans in coffee products (Becalski et al. 2010, 2016; Fromberg et al. 2014). Furthermore, the reported presence of different furan derivates in ready-to-eat baby food products indicates, that the same might also apply for infants and toddlers (Habibi et al. 2013; Shen et al. 2016; Knutsen et al. 2017). At present, the EFSA is unable to conclusively assess the extent to which DMF contributes to overall furan exposure, since the available data on DMF occurrence in food is still limited to date. Nevertheless, DMF amounts of 32–466 µg/kg and 69.4–230.3 µg/kg were determined in coffee and baby food, respectively (Habibi et al. 2013; Fromberg et al. 2014). Furthermore, DMF was detected in canned foods, roasted nuts, and Rooibos tea, while only minor amounts were present in breakfast cereals (Habu et al. 1985; Alasalvar et al. 2003; Fromberg et al. 2014; Lipinski et al. 2024). In addition to dietary intake, tobacco smoke represents a major source of human DMF exposure, with DMF is serving as sensitive biomarker in epidemiological studies (Jia et al. 2014). Therefore, it is reasonable to assume that DMF may contribute to overall furan exposure.

**Fig. 1:**
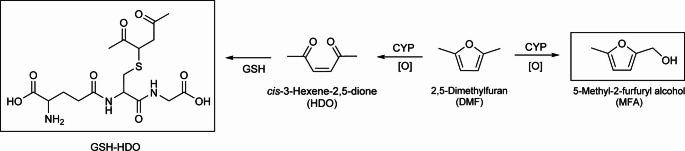
Investigated phase-I metabolites of the heat-induced contaminant DMF. CYP-mediated epoxidation of DMF results in the formation of the reactive *cis*-enedial metabolite HDO after intramolecular rearrangement. To deduce the in vitro formation kinetics, the reactive HDO was scavenged with GSH, resulting in the formation of the primary GSH-HDO conjugate. Contrary to its potential metabolic activation, CYP-catalysed side-chain hydroxylation of DMF results in the formation of the primary alcohol MFA

It is generally accepted, that the primary hepatotoxic effect of furan is attributed to cytochrome P450 (CYP)-mediated epoxidation of the aromatic double bond, resulting in the formation of the metabolite *cis*-butene-1,4-dial (BDA) (Chen et al. 1995). This highly reactive *cis*-enedial intermediate can subsequently form adducts with various cellular nucleophiles, including DNA (Byrns et al. 2002, 2004; Peterson et al. 2005, 2006; Karlstetter & Mally 2020; Kremer et al. 2023; Kalisch et al. 2024). An analogue biotransformation was also demonstrated for different methylfurans and various furan-containing compounds, indicating that CYP-mediated epoxidation is the main pathway for potential metabolic activation of molecules containing a furan ring as structural feature (Ravindranath et al. 1984, 1986; Ravindranath & Boyd 1985; Peterson 2013; Stegmüller et al. 2020; Schäfer et al. 2024; Bohlen et al. 2024). For DMF an equivalent biotransformation would result in the formation of *cis*-3-hexene-2,5-dione (HDO) as respective reactive metabolite (Fig. 1). As an α,β-unsaturated diketone, HDO is expected to be overall less reactive than BDA, however it is reasonable to assume that HDO can still react with cellular nucleophiles as Michael acceptor (Huffmann et al., 2016). Although HDO itself has not yet been detected in vitro or in vivo, corresponding *N*-acetyl lysine (NAL)-dimethylpyrrole-glutathione (GSH) adducts were identified in the bile of rats treated with DMF after ex vivo addition of NAL (Li et al. 2015). Formation of analogue pyrrole adducts was also observed in microsomal incubations fortified with different thiol- and primary amine-containing scavengers, supporting the assumption that HDO represents the main reactive metabolite of DMF formed by CYP-catalysed oxidation (Li et al. 2015, 2024; Wang et al. 2015). Despite these indications of a possible reactivity of HDO with cellular nucleophiles there is currently no evidence for its direct reactivity with DNA.

CYP2E1 was identified to exhibit the highest activity in oxidizing furan to BDA. However, several other CYP-isoforms, including CYP3A4, CYP2D6, CYP2B6, CYP2J2 and CYP1A2, also contribute to its metabolic activation, albeit with lower efficiency (Gates et al. 2012). Similar to furan, CYP2E1 is also the major catalyst for the metabolic activation of 2-methylfuran to its respective reactive metabolite 3-acetylacrolein (Schäfer et al. 2024). Due to the structural analogy, it is reasonable to assume that CYP2E1 is also the most active isoform in the potential metabolic activation of DMF, especially when considering that CYP2E1 generally has a high affinity for low-molecular compounds (Rendic 2002). Whether this assumption holds true and to what extent additional CYP-isoforms are involved in the metabolic activation of DMF has not yet been conclusively verified.

In contrast to their putative metabolic activation, hydroxylation of the alkyl moiety seems to be an alternative biotransformation pathway for methylfurans. As recently demonstrated by our group, this hypothesised side-chain hydroxylation, leading to formation of 5-methylfurfuryl alcohol (MFA) as an additional phase-I metabolite of DMF, is indeed catalysed by human microsomal enzymes (Fig. 1) (Bohlen et al. 2025). Although it is expected that this reaction is catalysed by hepatic CYPs, it is still unclear which isoforms are involved in the formation of MFA. Several publications on the metabolism of similar furan-containing alcohols, such as furfuryl alcohol (FFA) and hydroxymethylfurfural (HMF), have shown a rapid oxidation to the corresponding carboxylic acids, followed by efficient excretion, either directly or after conjugation with glycine (Nomeir et al. 1992; Germond et al. 1987; Heinzmann et al. 2015). Nevertheless, furan-containing alcohols such as MFA might on the other hand again be metabolically activated by sulfotransferase (SULT)-mediated sulfonation, potentially resulting in the formation of electrophilic sulfate conjugates. This pathway of biotransformation could already be demonstrated for FFA, including identification of respective DNA and amino acid adducts in vitro and in vivo (Monien et al. 2011, 2018, 2021; Sachse et al. 2014). It is currently unclear to what extent this putative form of biotransformation is also relevant for MFA or might contribute to the toxicity of DMF.

The main objective of this study was to monitor the parallel formation of the DMF phase-I metabolites HDO and MFA in human liver microsomes and deduce the respective in vitro formation kinetics. Due to its suggested reactivity, HDO was scavenged with GSH to subsequently quantitate the primary GSH-HDO conjugate by HPLC–ESI–MS/MS. In parallel we used an established GC–MS method to quantify MFA in the microsomal incubations. Subsequently, experiments with recombinant CYP-isoenzyme system were conducted to identify the CYP-isoforms involved in the formation of these two metabolites and to derive the respective formation kinetics for the individual CYP-isoforms.

## Materials and methods

### Chemicals

Unless otherwise specified, all chemicals and materials were obtained from Sigma Aldrich (Darmstadt, Germany), Merck (Darmstadt, Germany), or Thermo Fisher Scientific (Waltham, USA). Organic solvents were from VWR (Radnor, USA) and were of analytical grade. Pooled human liver microsomes from 20 male donors were purchased from Sigma Aldrich, while recombinant human Baculosome™ Plus Reagents (CYP1A2, CYP2B6, CYP2E1, CYP2D6, CYP3A4 and control Baculosomes™) were obtained from Thermo Fisher Scientific. l-Glutathione-(glycin-^13^C_2_,^15^N) trifluoroacetate salt (chemical purity ≥ 95%, isotopic purity ≥ 99% ^13^C, ≥ 98% ^15^N) was also ordered from Sigma Aldrich, and d_5_-furfuryl alcohol (d_5_-FFA**,** isotopic purity ≥ 99%) was obtained from CDN Isotopes (Quebec, Canada). 2,5-Dimethylfuran (purity 99%) used for synthesis of *cis*-3-hexene-2,5-dione (HDO) and as substrate for the in vitro experiments was also from Sigma Aldrich.

### Synthesis of *cis*-3-hexene-2,5-dione (HDO)

Synthesis of *cis*-3-hexene-2,5-dione (HDO) was performed by oxidation of DMF with dimethyldioxirane (DMDO). Therefore, 910 µl DMF were diluted in 11 ml of acetone. An equimolar amount of DMDO (90 ml, 8.55 mmol) was added dropwise over a period of 30 min and the reaction mixture was stirred for 45 min at room temperature. Afterwards, acetone was removed under reduced pressure. HDO was obtained as a brownish yellow liquid. The identity of HDO was confirmed by NMR spectroscopy (Fig. S1). Synthesis of DMDO was performed according to Adam et al. 1991.

^**1**^**H NMR** (400 MHz, **CDCl**_**3**_): δ/ppm: 6.30 (s, 1H, 3-H), 2.30 (s, 3H, 1-H).

### Synthesis of (2*S*)-2-amino-4-{[(1*R*)-1-[(carboxymethyl)carbamoyl]-2-[(2,5-dioxohexan-3-yl)sulfanyl]ethyl]carbamoyl}butanoic acid (GSH-HDO)

42.4 mg HDO (378.1 µmol) were diluted in 10 ml H_2_O. Subsequently, an equimolar amount reduced l-glutathione (116.2 mg, 378.1 µmol) was added and the reaction mixture was stirred for 2 h at room temperature. The reaction product was purified by preparative HPLC on a Synergi Polar-RP column, (4 µm, 10 × 250 mm, Phenomenex, Aschaffenburg, Germany) using an isocratic solvent composition of 100% H_2_O and a flow of 10 ml/min (t_R_ GSH-HDO: 7.6 min). Afterwards, the solvent was removed by lyophilization and GSH-HDO was obtained as a colourless solid. The identity of GSH-HDO was confirmed by NMR-spectroscopy (Fig. S2–S7) and exact mass measurement (timsTOF Pro, Bruker, Billerica, USA). A relative purity of 97% was determined utilizing the ^1^H- NMR-spectroscopy.

**Exact mass**: *m/z:* 420.145233* (*predicted *m/z*: 420.143511).

^**1**^**H NMR** (400 MHz, **D**_**2**_**O**) δ/ppm: 4.56 (td, ^*3*^* J* = 8.8, 5.3 Hz, 1H, 6-H), 3.95 (s, 2H, 15-H), 3.91–3.84 (m, 1H, 8-H), 3.81 (t, ^*3*^* J* = 6.3 Hz, 1H, 2-H), 3.30–3.22 (m, 1H, 9-H), 3.09–2.94 (m, 2H, 9-H/7-H), 2.88–2.77 (m, 1H, 7-H), 2.58–2.46 (m, 2H, 4-H), 2.36 (dd, ^*3*^* J* = 1.9, 2.2 Hz, 3H, 13-H), 2.21 (s, 3H, 11-H), 2.15 (dd, ^*3*^* J* = 14.2, 7.3 Hz, 2H, 3-H).

^**13**^**C NMR** (101 MHz, **D**_**2**_**O**) δ/ppm: 211.59, 208.79, 174.66, 173.53, 172.10, 172.05, 53.73, 52.97, 47.37, 46.97, 44.09, 41.50, 31.17, 29.05, 26.76, 26.00.

### Synthesis of (2*S*)-2-amino-4-{[(1*R*)-1-{[(carboxy(^13^C_2_)methyl](^15^N)carbamoyl}-2-[(2,5-dioxohexan-3-yl)sulfanyl]ethyl]carbamoyl}butanoic acid (^15^N^13^C_2_-GSH-HDO)

The synthesis of ^15^N^13^C_2_-GSH-HDO was performed analogue to the synthesis of GSH-HDO reported above. To maximize overall yield, 10 mg ^15^N^13^C_2_-l-glutathione (23.57 µmol) dissolved in 1 ml H_2_O were mixed with a fivefold excess of HDO (13.21 mg, 117.85 µmol). After incubation for 2 h at room temperature, the reaction product was purified by analytical HPLC on a Synergi Hydro-RP column (4 µm, 3 × 150 mm, Phenomenex) using an isocratic solvent composition of 85% H_2_O and 15% acetonitrile (*v/v*) and a flow of 0.8 ml/min (t_*R*_^15^N^13^C_2_-GSH-HDO: 1.7 min). The identity of ^15^N^13^C_2_-GSH-HDO was confirmed by the characteristic retention time and *m/z* of the precursor- and corresponding product-ions using HPLC–ESI–MS/MS (Table 1). Quantification was performed by HPLC–ESI–MS/MS using a calibration curve of the non-labelled GSH-HDO.

**Table 1:** Compound specific ESI^+^-MS/MS-parameters for GSH-HDO and the internal standard ^15^N^13^C_2_-GSH-HDO

Compound	t_*R*_ [min]	Precursor ion [*m/z*]	Product ion [*m/z*]	DP [V]	EP [V]	CEP [V]	CE [V]	CXP [V]
GSH-HDO	4.8	420.193	**273.100**	51	11	20	21	24
			170.300				33	4
			127.000				37	4
^15^N^13^C_2_-GSH-HDO	4.8	423.151	276.200	56	8	22	21	24
			**170.200**				33	4
			126.900				37	4

Depicted are respective retention times (t_*R*_), precursor ions, product ions, declustering potentials (DP), entrance potentials (EP), cell entrance potentials (CEP), collision energies (CE) and cell exit potentials (CXP). Respective quantifiers are bold.

### Microsomal incubations

Microsomal incubations were performed in 100 mM potassium phosphate buffer (pH 7.4) with a final protein concentration of 1 mg/ml. The NADPH-generating system consisted of 1 mM NADP^+^, 5 mM glucose-6-phosphate as well as 0.5 U/ml glucose-6-phosphate dehydrogenase. Additionally, the reaction mixture contained 3 mM magnesium chloride as well as 4 mM glutathione. After preincubation for 5 min at 37 °C, reactions were initiated by addition of varying concentrations DMF dissolved in acetonitrile (1% organic solvent) and incubated for 30 min at 37 °C under constant shaking. After incubation, the sample was split (1:1) and the reaction quenched by addition of an equal volume of either 20% trichloroacetic acid (TCA, *w/v*) containing 4 µM ^15^N^13^C_2_-GSH-HDO (for HPLC–ESI–MS/MS) or saturated ammonium sulfate solution containing 10 µM d_5_-FFA (for GC–MS), respectively. Subsequently, the samples were centrifuged (10 min, 17.000 g, 4 °C) and the supernatants transferred to new reaction tubes. Controls included incubations performed with heat-inactivated microsomes (5 min, 95 °C), without addition of the NADPH-generating system, and vehicle controls.

### Incubations with recombinant human CYP-isoenzyme systems

To investigate metabolite formation in Baculosome™ plus isoenzyme systems (CYP1A2, CYP2B6, CYP2E1, CYP2D6, CYP3A4) a comparable experimental setup with slight modifications was used. Reactions were performed in 100 mM potassium phosphate buffer (pH 7.4) using a final enzyme concentration of 100 pmol CYP/ml. Again, the reaction mixture contained 4 mM glutathione and 3 mM MgCl_2_. The composition of the NADPH-generating system was adjusted slightly to 1.3 mM NADP^+^, 3.3 mM glucose-6-phosphate, and 0.4 U/ml glucose-6-phosphate dehydrogenase. The reaction mixture was preincubated for 5 min at 37 °C. Afterwards, reactions were initiated by addition of DMF dissolved in acetonitrile (1% organic solvent). Incubations were performed for 20 min at 37 °C and quenched as described prior. Control Baculosomes™ devoid of recombinant human CYP-isoforms were included as negative controls.

### Sample preparation and quantification of MFA by GC–MS

Sample preparation and quantification of the phase-I metabolite MFA in microsomal and isoenzyme incubations were performed according to Bohlen et al. 2025. In brief, supernatants were acidified with 10 µl H_3_PO_4_ (1 M) and subsequently extracted three times with 150 µl methyl *tert*-butyl ether (MTBE). Afterwards, 100 µl acetonitrile were mixed with the combined organic extracts and MTBE was fully evaporated under argon atmosphere. Furthermore, 50 µl acetonitrile as well as 50 µl *N,O*-bis(trimethylsilyl)trifluoroacetamide (BSTFA) were added to the remaining 100 µl acetonitrile and the sample was subsequently derivatized for 20 min at 70 °C. Quantification of MFA was performed with an Agilent 7890 A GC system coupled to an Agilent 5975C mass spectrometer (Agilent Technologies, Santa Clara, USA). Samples were separated on a HP-5-MS fused silica capillary column (30 m × 0.25 mm x 0.25 µm, Agilent Technologies, Santa Clara, USA) under constant pressure of 11 psi, using helium as carrier gas. MFA (*m/z*: 95.1, 184.1, 169.1) and the internal standard d_5_-FFA (*m/z*: 86.1, 160.1) were monitored in the selected ion monitoring (SIM) mode at an electron energy (EI) of 70 eV. Retention times for MFA and d_5_-FFA were 5.55 min and 4.31 min, respectively. The temperature program, detailed method description and validation are available in Bohlen et al. 2025. Data was monitored and evaluated using MassHunter Workstation 10.0.368 (Agilent Technologies, Santa Clara, USA).

### Quantification of GSH-HDO by HPLC–ESI–MS/MS

Quantification of GSH-HDO in microsomal supernatants was conducted using an Agilent 1200 HPLC system (Agilent Technologies, Santa Clara, USA) coupled to an API 3200 Triple Quad mass spectrometer (AB SCIEX, Darmstadt, Germany). Samples were separated on a Synergi Hydro-RP column (4 µM, 150 × 3 mm, 80 Å, Phenomenex) using H_2_O with 0.1% formic acid (*v/v*) as solvent A and acetonitrile with 0.1% formic acid (*v/v*) as solvent B with a flow rate of 0.6 ml/min. Initial solvent composition of 95% A and 5% B was held for 4 min. Subsequently, solvent composition changed to 75% A and 25% B over the course of 0.1 min, followed by a linear gradient to 33% A and 67% B over 6.9 min. Afterwards solvent composition changed to 5% A and 95% B over 0.05 min and was held for 3 min. After returning solvent composition to initial conditions, reequilibration was performed for 5 min. Injection volume was 5 µl.

MS analysis was performed with positive electron spray ionization (ESI^+^) with a source temperature of 550 °C and ion spray voltage of 4000 V. Nitrogen served as curtain gas (50 psi), nebulizer gas (45 psi) and heater gas (60 psi). The mass spectrometer was operated in multiple reaction monitoring (MRM) mode. Compound specific ESI^+^-MS/MS parameters are presented in Table 1.

Calibration was performed in a concentration range of 0.05–100 µM GSH-HDO with 2 µM ^15^N^13^C_2_-GSH-HDO as internal standard and showed a linearity of *R*^2^ > 0.99. All standards were prepared in phosphate buffer mixed with 20% TCA (*w/v*) in a ratio of 1:1. Method precision was determined as coefficients of variation determined after five injections for two calibration levels of 0.1 and 50 µM (intraday repeatability) and repeated analysis of these standards over a period of five days (interday repeatability). Coefficients of variation for intra- and interday repeatability were determined to be 1.7–3.2% and 1.0–2.3%, respectively. Limit of detection (LOD), defined as signal to noise ratio of  ≥ 3 was 10 nM, while limit of quantification (LOQ), defined as signal to noise ratio of  ≥ 10 was 50 nM. Recovery rates of 99.7 ± 3.0% and 109.2 ± 4.3% were determined in heat inactivated human liver microsomes using two GSH-HDO concentrations (2 µM and 30 µM). Data was monitored and evaluated using Analyst 1.7.2 (Agilent Technologies, Santa Clara, USA).

### Data evaluation and curve fitting

To analyse the in vitro formation kinetics of the here investigated metabolites, the respective mean turnover rates were calculated from the absolute concentrations, determined after the end of the incubation period. For microsomal experiments, the turnover rates were expressed as pmol_metabolite_ / (µg_Protein_ × min), while turnover rates derived from experiments with recombinant CYP-isoenzyme systems were expressed as pmol_metabolite_ / (pmol_CYP_ × min). Data was fitted according to the Michaelis–Menten model (*v* = *V*_*max*_ × *c*_DMF_ / [*K*_*m*_ + *c*_DMF_]) with corresponding 95% confidence intervals, using Levenberg Marquardt algorithm and weighting type of 1 / σ^2^. Data evaluation and curve fitting was performed using Origin 2023 (OriginLab, Northhampton, USA).

## Results

By scavenging the reactive DMF metabolite HDO with GSH, it was possible to deduce HDO formation kinetics by quantification of the formed GSH-HDO adduct in human liver microsomes and recombinant CYP-isoenzyme systems using HPLC–ESI–MS/MS. Parallel quantification of the formed MFA using GC–MS enabled direct comparison of the formation kinetics of the two DMF phase-I metabolites in the in vitro systems used in this study.

### HDO and MFA formation kinetics in human liver microsomes

**Fig. 2:**
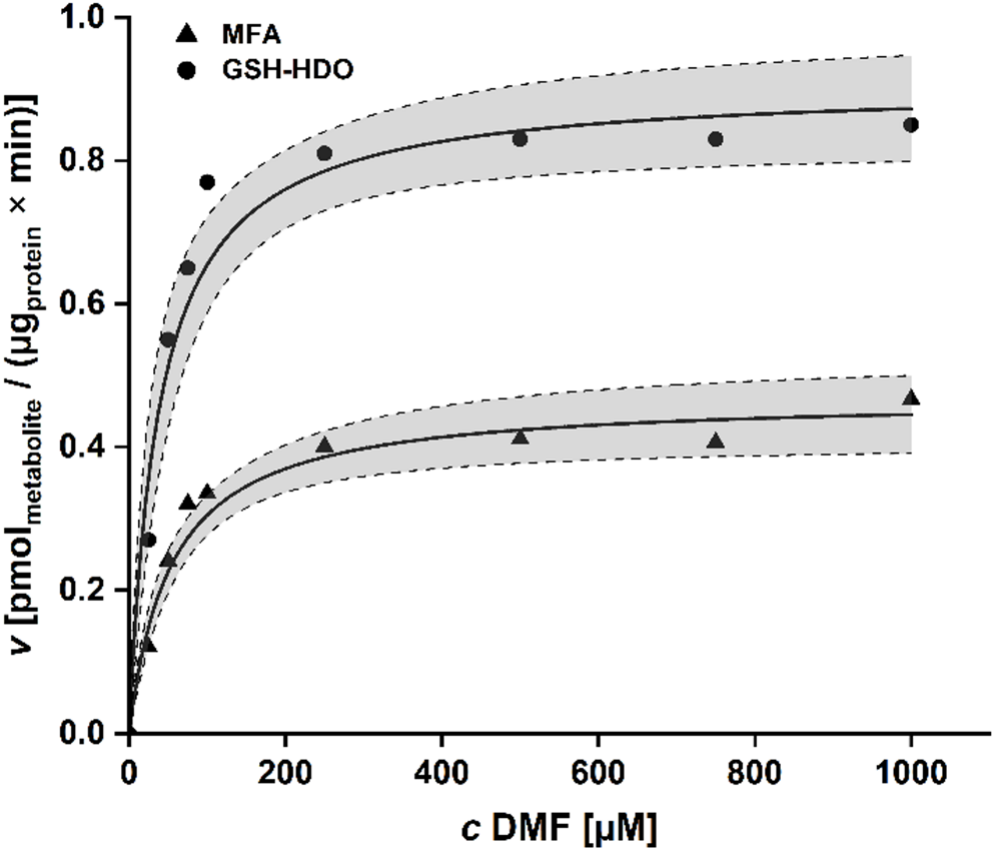
GSH-HDO and MFA formation kinetics in human liver microsomes. Data is depicted as mean turnover rate determined from three independent experiments (*n* = 3) and was fitted according to the Michaelis–Menten model. Respective 95% confidence intervals are indicated in grey.. Kinetic parameters for the formation of GSH-HDO and MFA in human liver microsomes derived from the fitted data: GSH-HDO: *K*_*m*_ ^*app*^ = 38.43 ± 8.24 µM, *V*_*max *_^*app*^: 0.91 ± 0.04 pmol / (µg × min); MFA: *K*_*m*_ ^*app*^ = 53.93 ± 8.91 µM, *V*_*max *_^*app*^: 0.47 ± 0.03 pmol / (µg × min)

The concentration-dependent formation of both, MFA and GSH-HDO was observed in human liver microsomes and followed classical Michaelis–Menten kinetics (Fig. 2). Calculated apparent Michaelis constants (*K*_*m*_^*app*^) for HDO (*K*_*m*_^*app*^: 38.43 ± 8.24 µM) and MFA (*K*_*m*_^*app*^: 53.93 ± 8.91 µM) formation in human liver microsomes were overall comparable, while apparent maximum reaction rates (*V*_*max *_^*app*^) for HDO (*V*_*max *_^*app*^: 0.91 ± 0.04 pmol / (µg × min) significantly exceeded maximum reaction rates of MFA (*V*_*max *_^*app*^: 0.47 ± 0.03 pmol / (µg × min). Respective controls consisted of incubations with heat inactivated microsomes, in absence of the NADPH-generating system and respective vehicle controls. None of the controls showed significant metabolite formation (Fig. S8 & S10).

### HDO and MFA formation kinetics in recombinant human CYP-isoenzyme systems

**Fig. 3:**
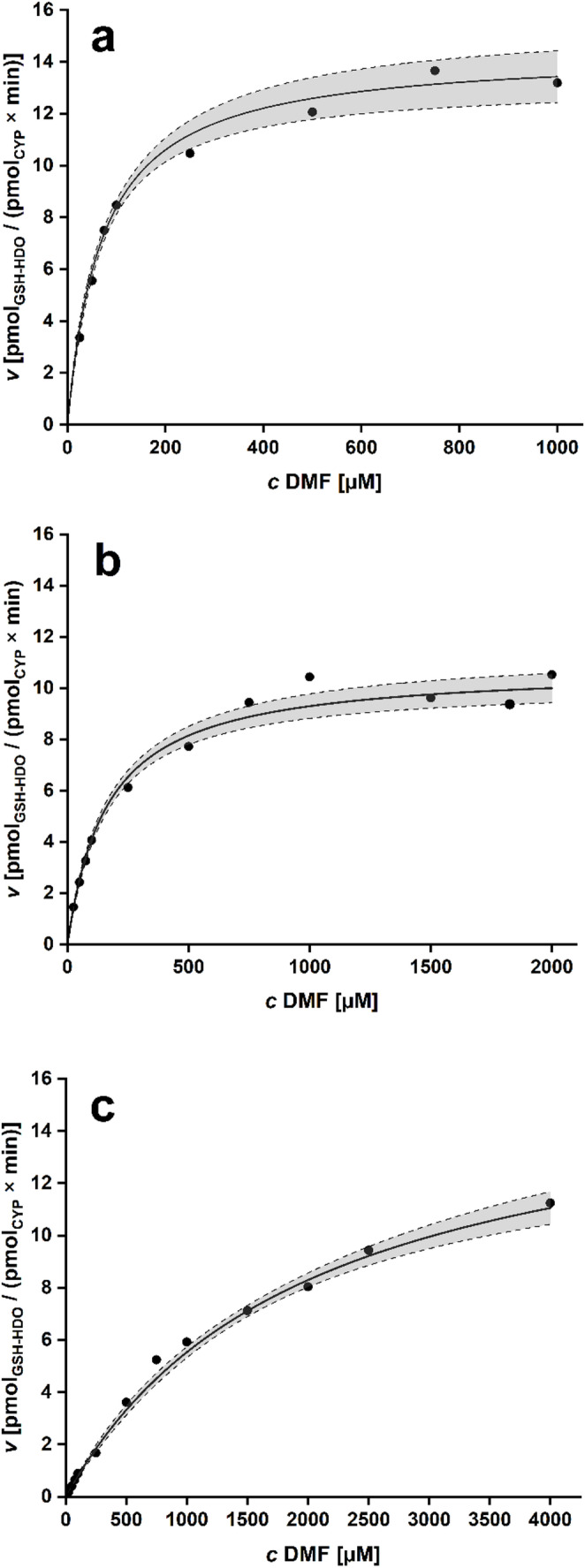
Formation kinetics of GSH-HDO formed in human Baculosome™ CYP2E1 (**a**), CYP3A4 (**b**), and CYP2D6 (**c**) isoenzyme systems. Data is depicted as mean turnover rate determined from three independent experiments (*n* = 3) and was fitted according to the Michaelis–Menten model. Respective 95% confidence intervals are indicated in grey. Respective kinetic parameters for the formation of GSH-HDO were determined for the individual CYP-isoforms: CYP2E1 **(a)** *K*_*m*_ ^*app*^ = 71.43 ± 5.71 µM, *k*_*cat *_^*app*^ = 14.49 ± 0.52 pmol / (pmol_CYP_ × min); CYP3A4 **(b)** *K*_*m*_ ^*app*^ = 163.82 ± 9.18 µM*, k*_*cat *_^*app*^ = 10.82 ± 0.31 pmol / (pmol_CYP_ × min); CYP2D6 **(c)** *K*_*m*_ ^*app*^ = 1991.26 ± 191.62 µM, *k*_*cat *_^*app*^ = 16.56 ± 0.92 pmol / (pmol_CYP_ × min)

Three recombinant human CYP-isoforms, namely CYP2E1, CYP3A4, and CYP2D6 were identified to catalyse metabolic activation of DMF to the reactive metabolite HDO. On the other hand, no metabolite formation could be observed in CYP1A2 and CYP2B6 as well as control systems (Fig. S9). Concentration dependent formation of GSH-HDO was most pronounced for CYP2E1, showing the lowest *K*_*m*_ ^*app*^ of 71.43 ± 5.71 µM and highest catalytic constant (*k*_*cat *_^*app*^) of 14.49 ± 0.52 pmol / (pmol_CYP_ × min) (Fig. 3a). CYP3A4 contribution to HDO formation was less pronounced but still significant. Calculated *k*_*cat *_^*app*^ of 10.82 ± 0.31 pmol / (pmol_CYP_ × min) was slightly lower, however determined *K*_*m*_ ^*app*^ of 163.82 ± 9.18 µM was considerably higher compared to CYP2E1 (Fig. 3b). While the determined *k*_*cat *_^*app*^ of 16.56 ± 0.92 pmol / (pmol_CYP_ × min) for HDO formation in the CYP2D6 system was overall comparable to CYP2E1, determined *K*_*m*_ ^*app*^ of 1991.26 ± 191.62 µM for the reaction was exceptionally high (Fig. 3c).

**Fig. 4:**
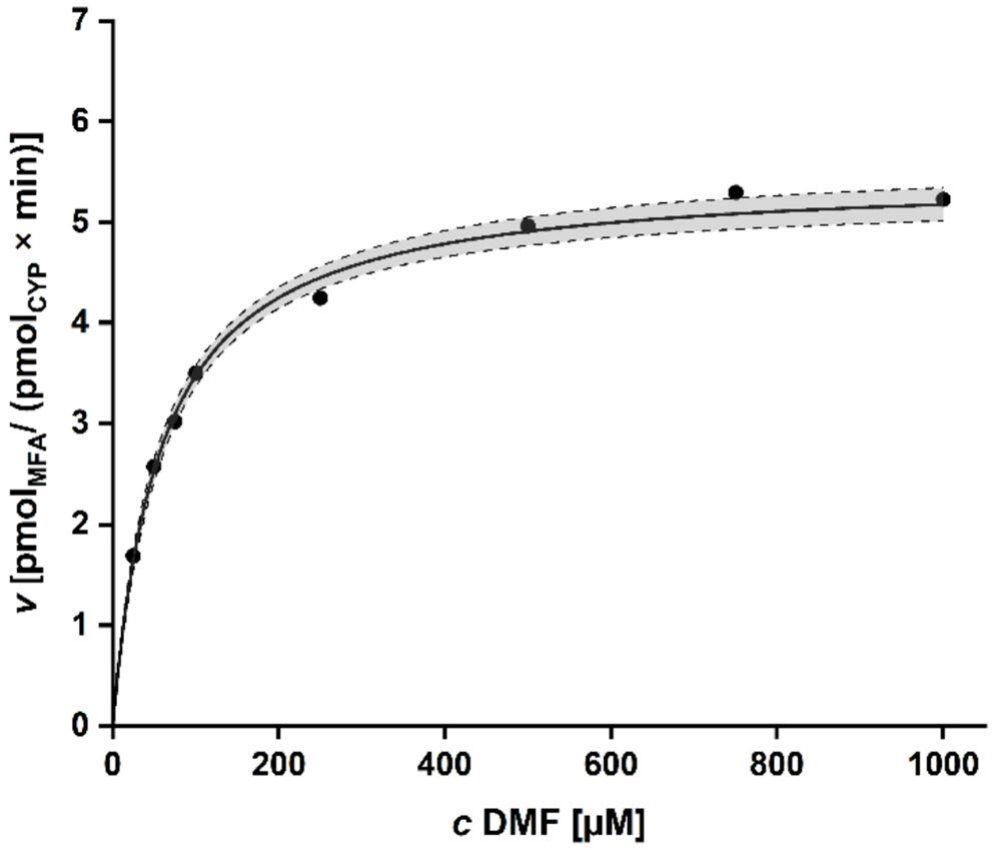
Formation kinetics of MFA in human CYP2E1 Baculosome™ isoenzyme systems. Data is presented as mean turnover rate determined from three independent experiments (*n* = 3) and was fitted using the Michaelis–Menten model. The corresponding 95% confidence interval is indicated in grey. A *K*_*m*_ ^*app*^ of 57.61 ± 2.86 µM and *k*_*cat *_^*app*^ of 5.47 ± 0.08 pmol / (pmol_CYP_ × min) for the reaction were calculated from the fitted data

The formation of MFA was exclusively catalysed by CYP2E1 (Fig. 4). For the other selected human CYP-isoforms and the controls no formation of MFA could be observed (Fig. S11). With a calculated *k*_*cat *_^*app*^ of 5.47 ± 0.08 pmol / (pmol_CYP_ × min), MFA formation by CYP2E1 was considerably slower compared to the formation of HDO. However, determined *K*_*m*_ ^*app*^ of 57.61 ± 2.86 µM for the reaction was overall comparable to HDO formation in the CYP2E1 system (Fig. 3a).

## Discussion

The toxicity of furan is expected to be based on its CYP-mediated metabolic activation to the reactive *cis*-enedial metabolite BDA, which can form adducts with various cellular nucleophiles. An analogous biotransformation of the alkylfuran DMF is anticipated to results in formation of the reactive and potentially toxic *cis*-enedione metabolite HDO.

Here, HDO formed after biotransformation of DMF was scavenged with GSH. Using this approach, it was possible to quantify the primary GSH-HDO adduct by HPLC–ESI–MS/MS and deduce HDO formation kinetics in different in vitro systems such as microsomes and CYP-isoenzyme systems. Contrary to this metabolic pathway of activation, DMF is also subject to CYP-catalysed side-chain hydroxylation, leading to the formation of the primary alcohol MFA (Bohlen et al. 2025). Using the here established HPLC–ESI–MS/MS method to quantitate GSH-HDO together with a GC–MS method for quantification of MFA, it was possible to monitor and compare HDO and MFA formation kinetics in vitro.

In line with previous results, the parallel formation of both DMF phase-I metabolites was observed in microsomal incubation experiments. Although calculated Michaelis constants for both reactions are roughly similar (*K*_*m*_ ^*app*^ _HDO_ = 38.43 ± 8.24 µM; *K*_*m*_ ^*app*^_MFA_ = 53.93 ± 8.91 µM), the maximum reaction rate for the formation of HDO (*V*_*max *_^*app*^: 0.91 ± 0.04 pmol / (µg × min)) significantly exceeds the maximum rate for the formation of MFA (*V*_*max *_^*app*^: 0.47 ± 0.03 pmol / (µg × min)). The GC–MS method used in this study also allows for the quantification of MFF and MFC as potential oxidation products of MFA. Consistent with previously published results, no significant formation of 5-methyl-2-furfural (MFF) or 5-methyl-2-furancarboxylic acid (MFC) was detected in experiment with human liver microsomes or isoenzyme systems, indicating that a potential further oxidation of MFA is not catalysed by hepatic CYPs (Bohlen et al. 2025). Thus, our kinetic data demonstrates that metabolic activation of DMF to the reactive metabolite HDO is the favoured pathway of biotransformation in human liver microsomes compared to the alternative side-chain hydroxylation (Fig. 1). Especially at submicromolar DMF concentrations, far below the determined *K*_*m*_, and thus much closer to a realistic human exposure scenario, this difference in formation rates is expected to be more pronounced. It can therefore be assumed that at physiologically relevant concentrations CYP-mediated epoxidation of DMF and formation reactive metabolite HDO may represent the favoured pathway of hepatic biotransformation.

Peterson and colleagues reported a *K*_*m*_ of 37.6 ± 4.3 µM and *V*_*max*_ of 2.5 ± 0.1 pmol / (µg × min) for the metabolic conversion of furan to its respective reactive metabolite BDA after trapping with GSH in rat microsomal preparations (Peterson et al. 2005). In contrast to the formation of HDO, reported BDA formation rate was higher but in the same order of magnitude, while *K*_*m*_ values for both reactions were overall comparable. Gates et al., (2012) reported a mean BDA formation rate of 1.3 pmol / (µg × min) after incubation of human microsomes with 50 µM furan, exceeding the here determined HDO formation rate of 0.9 pmol / (µg x min). While this data suggests that the metabolic activation of DMF may generally occur at a slightly slower rate than furan, it appears to be overall comparable, especially when considering species differences and the here demonstrated competition between metabolic activation and side-chain hydroxylation for the biotransformation of DMF in microsomal preparations.

Five recombinant human CYP-isoforms, namely CYP1A2, CYP2B6, CYP2E1, CYP2D6, CYP3A4, were selected to investigate which hepatic CYP-isoforms mediate the phase-I biotransformation of DMF and monitor the respective formation kinetics. The selection was based on literature data regarding the CYP-mediated metabolism of furan and various furan-containing compounds and included the isoforms that contribute most to the hepatic biotransformation of chemicals (Rendic 2002; Peterson 2013; Rendic & Guengerich 2021; Tian et al. 2022). The obtained kinetic data clearly shows that CYP2E1 is the major contributor to the metabolic activation of DMF and formation of the reactive metabolite HDO (*K*_*m*_ ^*app*^ = 71.43 ± 5.71 µM, *k*_*cat *_^*app*^ = 14.49 ± 0.52 pmol / (pmol_CYP_ × min); Fig. 3a). As CYP2E1 is also the main isoform that activates furan and 2-methylfuran and on the other hand mediates the biotransformation of several low-molecular-weight chemicals and drugs, this observation is plausible (Rendic 2002; Gates et al. 2012; Rendic & Guengerich 2021; Schäfer et al. 2024). Consistent with the results obtained in human liver microsomes, CYP2E1-mediated metabolic activation of DMF appears to be slower than that of furan, especially pronounced at low substrate concentrations. This becomes particularly evident when comparing the reported apparent catalytic efficiency (*E*_*cat*_^*app*^) for the metabolic activation of furan in CYP2E1 supersomes (*E*_*cat*_^*app*^: 3.9 × 10^–1^ min^−1^ × µM^−1^) with the here investigated metabolic activation of DMF (*E*_*cat*_^*app*^: 2.0 × 10^–1^ min^−1^ × µM^−1^) (Peterson et al. 2005). A markedly higher *K*_*m*_^*app*^ (163.82 ± 9.18 µM) and slightly lower *V*_*max*_^*app*^ (10.82 ± 0.31 pmol / (pmol_CYP_ × min) were determined for the CYP3A4-catalysed HDO formation (Fig. 3b), resulting in a significantly lowered catalytic efficiency (*E*_*cat*_^*app*^: 6.6 × 10^–2^ min^−1^ × µM^−1^). Our data suggest that CYP3A4 may still contribute significantly to hepatic HDO formation, especially when considering that hepatic CYP3A4 levels are usually higher than those of CYP2E1 (Shimada et al. 1994; Sadler et al. 2016; Liu et al. 2014). In addition, CYP2D6 was also identified to catalyse the metabolic activation of DMF (Fig. 3c), however the calculated *K*_*m*_^*app*^ (1991.26 ± 191.62 µM) for the reaction was exceptionally high, while the *k*_*cat*_^*app*^ (16.56 ± 0.92 pmol / (pmol_CYP_ × min) was overall comparable to CYP2E1 (14.49 ± 0.52 pmol / (pmol_CYP_ × min). Due to the low catalytic efficiency of CYP2D6 (*E*_*cat*_^*app*^: 8.3 × 10^–3^ min^−1^ × µM^−1^), only a minimal contribution to the formation of HDO can be expected at realistic DMF concentrations in contrast to CYP2E1 and CYP3A4. However, contribution of CYP2D6 may be relevant for some individuals, since major interindividual variations in CYP2D6 activity are well known (Ingelman-Sundberg 2005; Neafsey et al. 2009; Zhou et al. 2009). Consequently, there might be some individuals that are more susceptible to CYP2D6-mediated HDO formation. Although a minor contribution of CYP2B6 and CYP1A2 to the metabolic activation of furan has been reported, this could not be confirmed for DMF here (Gates et al. 2012).

Contrary to its metabolic activation, the side-chain hydroxylation of DMF was exclusively catalysed by CYP2E1. In line with the results obtained from microsomal incubations, this reaction proceeded significantly less efficient (*E*_*cat*_^*app*^: 9.5 × 10^–2^ min^−1^ × µM^−1^) compared to the formation of HDO (*E*_*cat*_^*app*^: 3.9 × 10^–1^ min^−1^ × µM^−1^). This further underlines the conclusion that aromatic epoxidation is the main route of hepatic biotransformation of DMF, particularly at concentrations below the determined *K*_*m*_ (Fig. 4). Since several publications indicate that the formed MFA is oxidized rapidly to the corresponding carboxylic acid and excreted efficiently, it is reasonable to assume that hydroxylation of DMF primarily represents a detoxification pathway, especially when compared to the formation of the reactive *cis*-enedial metabolite HDO (Germond et al. 1987; Nomeir et al. 1992; Heinzmann et al. 2015). However, as already demonstrated for furfuryl alcohol, furan-containing alcohols including MFA might serve as substrates for sulfotransferases (Monien et al. 2011, 2018, 2021; Sachse et al. 2014). Since the potentially formed sulfate conjugates are highly electrophilic and may therefore react with cellular nucleophiles, it is possible that the hydroxylation of DMF and subsequent sulfonation may at least partially represent a further pathway for metabolic activation. However, to what extend MFA formation might contribute to the toxicity of DMF via this hypothesised metabolic conversion is still unknown and under investigation.

## Conclusion

In summary our data demonstrates that the putative metabolic activation and the formation of the reactive metabolite HDO represents the favoured biotransformation pathway for DMF in human liver microsomes, despite the concurrent formation of MFA through side-chain hydroxylation. Furthermore, CYP2E1 is expected to be the main contributor to the hepatic biotransformation of DMF, especially in the context of realistic exposure scenarios. Although the hypothesized metabolic activation of DMF appears to occur overall less efficient than that of furan, kinetic parameters for both reactions seem to be overall comparable. Due to its frequent presence in food and its comparable metabolism, DMF may therefore contribute to overall furan exposure, increasing the risk of furan-related adverse effects for consumers, especially vulnerable groups such as infants and toddlers. However, to what extent the biotransformation of DMF to HDO contributes to its potential toxicity or if alternative mechanisms are more relevant in this context still needs to be elucidated.

## Supplementary Information

Below is the link to the electronic supplementary material.

Supplementary Material 1.

## Abbreviations

BDA: *cis*-2-Butene-1,4-dial
BSTFA: *N,O*-Bis(trimethylsilyl)trifluoroacetamide
CYP: Cytochrome P450
DMDO: Dimethyldioxirane
DMF: 2,5-Dimethylfuran
*E*_*cat*_^*app*^: Apparent catalytic efficiency
EFSA: European Food Safety Authority
FFA: Furfuryl alcohol
d_5_-FFA: d_5_-Furfuryl alcohol
GSH: Glutathione
GSH-HDO: (2*S*)-2-Amino-4-{[(1*R*)-1-[(carboxymethyl)carbamoyl]-2-[(2,5-dioxohexan-3-yl)sulfanyl]ethyl]carbamoyl}butanoic acid
HDO: *cis*-3-Hexene-2,5-dione
HMF: Hydroxymethylfurfural
IARC: International Agency for Research on Cancer
*k*_*cat*_^*app*^: Apparent turnover rate
*K*_*m*_^*app*^: Apparent Michaelis constant
MFA: 5-Methylfurfuryl alcohol
MRM: Multiple reaction monitoring
MTBE: Methyl *tert*-butyl ether
^15^N^13^C_2_-GSH-HDO: (2*S*)-2-Amino-4-{[(1*R*)-1-{[(carboxy(^13^C_2_)methyl](^15^N)carbamoyl}-2-[(2,5-dioxohexan-3-yl)sulfanyl]ethyl]carbamoyl}butanoic acid
NAL: *N*-Acetyl lysine
NTP: National toxicology program
SIM: Selected ion monitoring
SULT: Sulfotransferase
TCA: Trichloroacetic acid
*V*_*max*_^*app*^: Apparent maximum velocity
