# Supplementary material for: In vitro formation kinetics of two 2,5-dimethylfuran phase-I metabolites: cis-3-hexene-2,5-dione and 5-methylfurfuryl alcohol
Source: Arch Toxicol. 2026 May 20;100(9):3935–45. doi: 10.1007/s00204-026-04442-8 (PMC13454002; doi:10.1007/s00204-026-04442-8)
Supplement: Supplementary file 1 [file 204_2026_4442_MOESM1_ESM.docx]

**Supplementary information**

***In vitro* formation kinetics of two 2,5-dimethylfuran phase-I metabolites - *cis*-3-hexene-2,5-dione and 5-methylfurfuryl alcohol**

**Archives of Toxicology**

Jonas Appel, Carolin Kulosa, Nico Becker, Simone Stegmüller, Sullivan Sadzik, Elke Richling*

Department of Chemistry, Division of Food Chemistry and Toxicology, RPTU University Kaiserslautern-Landau (RPTU), Kaiserslautern, Germany

*Corresponding author

Email: elke.richling@chem.rptu.de

ORCID: 0000-0001-5746-8032

**Fig. S1:** ^1^H-NMR spectrum (400 MHz) of synthesized (3Z)-3-hexene-2,5-dione (HDO) in CDCl_3._

|  | ^1^H δ/ppm; ∫; m; J/Hz | ^13^C δ/ppm |
| --- | --- | --- |
| **1** | - | 174.66/173.53 |
| **2** | 3.81; 1 H; t; 6.4 | 53.73/52.97 |
| **3** | 2.15; 2 H; dd; 14.2/7.3 | 26.76/26.00 |
| **4** | 2.58–2.46; 2 H; m | 31.17 |
| **5** | - | 174.66/173.53 |
| **6** | 4.56; 1 H; td; 8.8/5.3 | 53.73/52.97 |
| **7** | 2.88–2.75, 1 H; m  3.09–2.94; 1 H; m | 41.50 |
| **8** | 3.91–3.84; 1 H; m | 47.37 |
| **9** | 3.30–3.22; 1 H; m  3.09–2.94; 1 H; m | 45.97/44.09 |
| **10** | - | 211.59 |
| **11** | 2.21; 3 H; s | 29.05 |
| **12** | - | 208.79 |
| **13** | 2.36; 3 H; d; 1.9 | 26.76/26.00 |
| **14** | - | 172.10/172.05 |
| **15** | 3.95; 2 H; s | 45.97/44.09 |
| **16** | - | 172.10/172.05 |

**Fig. S2:** Structure of GSH-HDO and assignment of the corresponding ^1^H- and ^13^C-NMR signals.

**Fig. S3:** ^1^H-NMR spectrum (400 MHz) of synthesized GSH-HDO in D_2_O after purification by preparative HPLC.

**Fig. S4:** ^13^C-NMR spectrum (101 MHz) of synthesized GSH-HDO in D_2_O after purification by preparative HPLC.

**Fig. S5:** HSQC-NMR spectrum of synthesized GSH-HDO in D_2_O after purification by preparative HPLC.

**Fig. S6:** H-H COSY-NMR spectrum of synthesized GSH-HDO in D_2_O after purification by preparative HPLC.

**Fig. S7:** HMBC-NMR spectrum of synthesized GSH-HDO in D_2_O after purification by preparative HPLC.

**Fig. S8:** Representative chromatograms of microsomal supernatants monitored by HPLC-ESI-MS/MS. Depicted are **(a)** a 10 µM calibration standard of GSH-HDO (t*_R_*: 4.8 min) and ^15^N^13^C_2_-GSH-HDO (t*_R_*: 4.8 min) and chromatograms monitored after **(b)** incubation of human liver microsomes with 1000 µM DMF, **(c)** incubation of heat inactivated human liver microsomes with 1000 µM DMF and **(d)** incubation of human liver microsomes without DMF for 30 min.

**Fig. S9:** Representative chromatograms of Baculosome™ supernatants monitored by HPLC-ESI-MS/MS. Depicted are chromatograms monitored after incubation of **(a)** hCYP2E1 and **(b)** control Baculosomes™ with 250 µM DMF for 20 min.

**Fig. S10:** Representative chromatograms of microsomal supernatants monitored by GC-MS. Depicted are **(a)** a 10 µM calibration standard of MFF (*m/z*: 110.1, 109.1; t*_R_*: 3.82 min, ), MFA (*m/z*: 95.1, 184.1, 169.1; t*_R_*: 6.12 min), MFC (*m/z*: 139.1, 183.1, 109.1; t*_R_*: 8.80 min) and d_5_-FFA (*m/z*: 86.1, 160.1; t*_R_*: 4.33 min) as well as chromatograms monitored after **(b)** incubation of human liver microsomes with 1000 µM DMF, **(c)** incubation of heat inactivated human liver microsomes with 1000 µM DMF and **(d)** incubation of human liver microsomes without DMF for 30 min.

**Fig. S11:** Representative chromatograms of Baculosome™ supernatants monitored by GC-MS. Depicted are chromatograms monitored after incubation of **(a)** hCYP2E1 and **(b)** control Baculosomes™ with 250 µM DMF for 20 min. MFA (*m/z*: 95.1, 184.1, 169.1, t*_R_*: 5.55 min), d_5_-FFA (*m/z*: 86.1, 160.1, t*_R_*: 4.31 min).
